# Standardized Elemental Composition Analysis of Graphene‐Related 2D Materials (GR2M) With SEM/EDS and XPS Works Reliably

**DOI:** 10.1002/smll.202511283

**Published:** 2026-03-09

**Authors:** Paul Mrkwitschka, Mario Sahre, Elena Corrao, Francesco Pellegrino, Beatriz Alonso, Amaia Zurutuza, Jörg Radnik, Vasile‐Dan Hodoroaba

**Affiliations:** ^1^ Division 6.1 Surface and Thin Film Analysis Federal Institute for Research and Testing (BAM) Berlin Germany; ^2^ Department of Chemistry and NIS Centre University of Torino Torino Italy; ^3^ Graphenea San Sebastián Spain

**Keywords:** graphene oxide flakes, impurities, O/C ratio, SEM/EDS, standard, XPS

## Abstract

Reliable quantification of the chemical composition of graphene‐related 2D materials (GR2M) as powders and liquid suspensions is a challenging task. Analytical methods such as X‐ray photoelectron spectroscopy (XPS), inductively coupled plasma mass spectrometry (ICP‐MS), thermogravimetric analysis (TGA) and Fourier transform infrared spectroscopy (FTIR) are recommended by standardization bodies. The specific parameters to be measured are also defined, e.g., the oxygen‐to‐carbon (O/C) atomic ratio, the trace metal impurities, or the functional groups. In this contribution, for the first time, results of a systematic study on the capability of energy‐dispersive X‐ray spectroscopy (EDS) at a scanning electron microscope (SEM) to reliably quantify the O/C ratio and impurities remained from the synthesis of selected GR2M are reported. The robustness of SEM/EDS analysis is verified for various measurement conditions (different excitations and EDS detectors) and the validity of the results is tested by comparison to the established XPS analysis. Moreover, an ionic liquid is used as a reference material for the quantification of the light elements such as C, N, O and F. The study clearly demonstrates the reliability of the fast and widely available SEM/EDS as a standard method for the quantification of the elemental composition of GR2M and generally of light materials.

## Graphical abstract

The elemental composition of a series of laboratory graphene oxide and reduced graphene oxide materials and a commercial graphene oxide material of high quality is analyzed with the standardless SEM/EDS quantification. XPS is used as a reference method. The quantification of light elements of both methods is validated by considering an ionic liquid sample as a reference material.

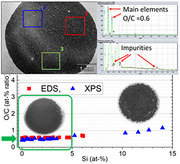

## 1 Introduction

Graphene‐related 2D materials (GR2M) supplied as flakes in powder form, dispersed in water or organic liquids, as additives in paints and cement, and as part of composites in components and inks are more and more available as commercial products [1, 2]. They are already used in optoelectronics, energy storage, chemical additives, and sensors. One of the most promising applications is flexible electronics [3]. To make these new products competitive with the established ones, it is necessary to assess and quantify, but also to improve the specific functional properties of the products made from GR2M. This is possible in turn by a complete understanding of the physico‐chemical properties of the GR2M products in their final form including the exact knowledge also of the starting materials down to the nanoscale [1, 2, 3, 4]. Well‐defined parameters and classification frameworks are necessary and required also by regulatory bodies for the corresponding commercial products entering the market [5, 6, 7, 8, 9, 10, 11]. Thus, the morphology and structure of the GR2M shall be expressed quantitatively through descriptors which are well‐defined in published (or under development) ISO standards such as particles/platelets/flakes size (lateral and thickness) and shape distribution, numbers of layers, layer alignment, level of disorder, estimated number fraction of graphene (G) or few‐layer graphene (FLG), the specific surface area of the powder containing G [5, 6, 7, 8, 9, 10, 11]. As far as the chemical composition of GR2M is concerned, the oxygen‐to‐carbon at‐% ratio, elemental composition, trace metal impurities and weight percentage of chemical species and the functional groups present are defined as parameters to be measured with standardized analysis methods [11]. Especially, the O/C ratio and functionalization tailor the properties of GR2M, and, herewith, determine the interaction between the GR2M and solvents and matrices [12, 13]. Following analytical methods: X‐ray photoelectron spectroscopy (XPS) as the central one, inductively coupled plasma mass spectrometry (ICP‐MS), thermogravimetric analysis (TGA) and Fourier transform infrared spectroscopy (FTIR) are recommended by the standardization bodies to be used for the chemical characterization of GR2M from powders and liquid dispersions. Included are also dedicated protocols for sample preparation, measurement and data analysis. It should be noticed that the protocols developed for GR2M should be possibly transferable to other 2D materials with similar morphologies (e.g., of platelet shape) or chemistry (containing a significant fraction of light elements), such as MXenes, 2D transition metal dichalcogenides (TMD), boron(carbo)nitrides, *etc*. Substantial pre‐standardization projects paving the way for the development of ISO standards and regulations are hosted under the VAMAS platform [14], with terminology projects being also part of [15, 16].

In this manuscript, for the first time, the capability of energy‐dispersive X‐ray spectroscopy (EDS) at a scanning electron microscope (SEM) to reliably quantify the light elements such as C, N, O and F, and with this the O/C ratio, and impurities remained from synthesis for selected GR2M is systematically tested. The electron probe microanalysis (EPMA) is an about 60 years old analytical technique able to quantify elements in solid matter. Conventionally, the quantification of the elemental composition with EPMA in the wavelength‐dispersive X‐ray (WDS) detection version or EDS at an SEM works reliably for elements with the atomic number of 11 and above [17, 18, 19, 20]. With the same quantification algorithms as decades ago, but with the technological development and establishment of modern thin‐film windows and silicon‐drift detectors (SDD), EDS with a SEM is meanwhile probably the widest available analytical method, being able to quantify standardless, and hence extremely fast the elemental composition in the micrometer range of solid matter also for light elements such as C, N, O and F. Particularly the development of compact, table‐top SEM/EDS instruments of increasing analytical quality is remarkable. Thus, one main objective of this study becomes obvious, namely, to systematically evaluate the performance of SEM/EDS as a high‐throughput method to be included into the standardized characterization workflow for fast and accurate analysis of the elemental composition of GR2M. The general expectation is that the measurement uncertainty for the light elements having X‐ray lines below 1 keV is significantly larger than that for the elements with K X‐ray lines at higher energies, i.e. with atomic numbers of 11 and above [17, 18, 19, 20]. Different graphene oxide (GO) materials, synthesized in the laboratory and of commercial provenance will be systematically analyzed with SEM/EDS comparatively to XPS as the already established method for the elemental analysis of GR2M for validation purpose [21, 22, 23].

The robustness of SEM/EDS elemental analysis on GR2M will be verified for various excitation conditions and different EDS detector manufacturers. Moreover, to check the accuracy of the quantification results of the light elements such as C, N, O and F obtained with SEM/EDS, but also with XPS, an ionic liquid with well‐known stochiometric elemental composition is considered to be benchmarked as a reference material [24, 25]. The suitability of this type of material as a reference material is extensively demonstrated in [26, 27]. It should be emphasized that SEM/EDS can provide ‘only’ elemental analysis and not chemical‐state information, so that it constitutes a suitable quantitative screening tool which shall be complementary to XPS, FTIR and Raman spectroscopy for functional group analysis.

## 2 Results and Discussion

### 2.1 Laboratory GO/rGO Materials

**FIGURE 1:**
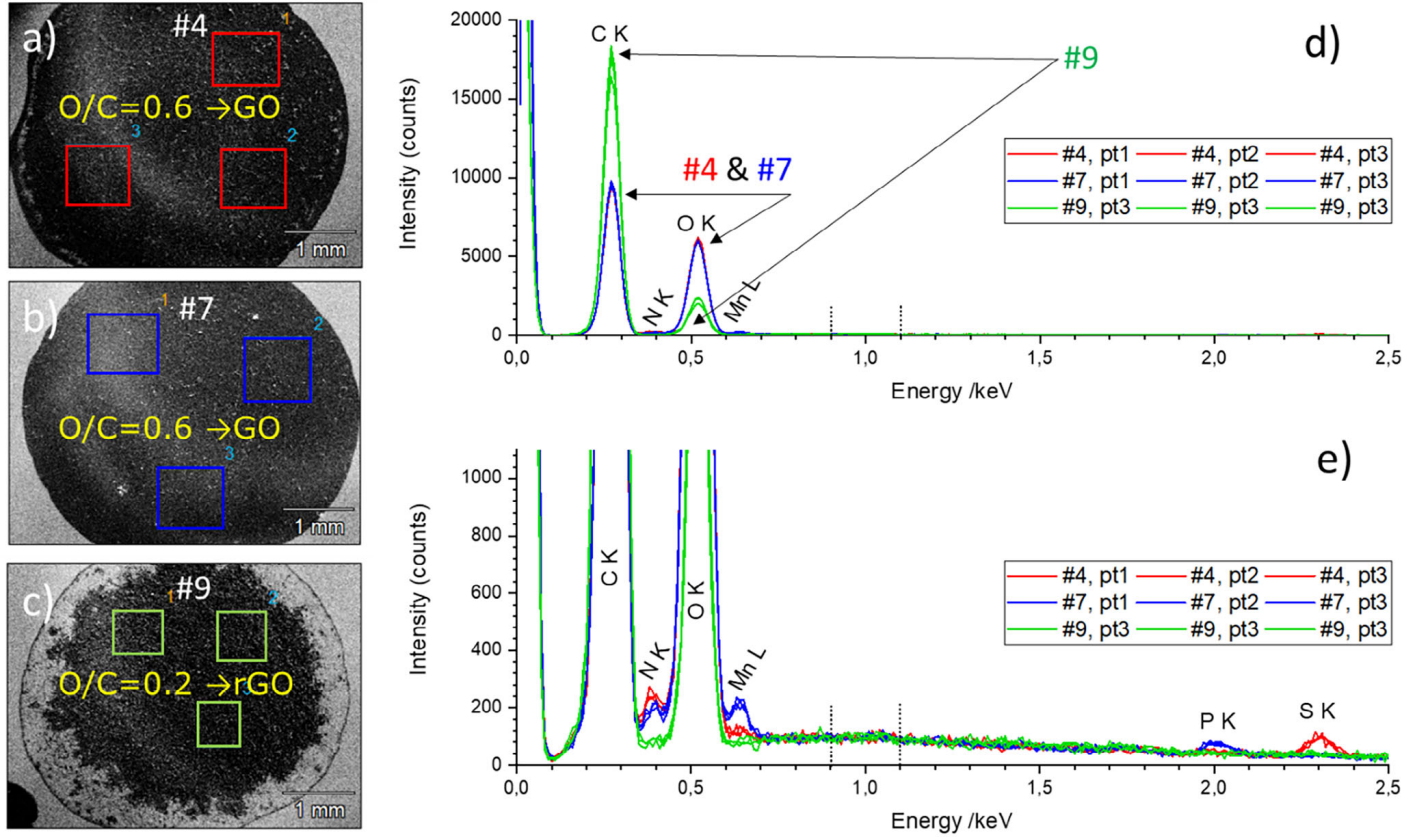
SEM micrographs and 5 kV EDS spectra of 3 different GR2M materials measured at three locations on each sample: a) #4 (GO), b) #7 (GO) and c) #9 (rGO), with 2 oxidation levels, i.e. O/C = 0.6 for samples #4 and #7 and O/C = 0.2 for sample #9 (d). Note the specific impurities detected in the different materials (e). All the spectra are normalized to the background intensity in the range 0.9 to 1.1 keV.

First, a series of three different GO materials with different oxidation levels synthesized in laboratory as part of the European project ACCORDs (https://accordsproject.com/) has been analyzed and standardless quantified with SEM/EDS. Details on the synthesis can be found in [26]. The SEM and EDS analysis results after deposition of the GO flake materials over suspension route onto a silicon substrate are showed in Figure 1. While for the first two materials, i.e. #4 and #7, the O/C at‐% ratio is 0.6, for the third material, i.e., #9, the corresponding O/C at‐% ratio is 0.2. These numbers are in close agreement with the recommendations of ISO TS 80004‐13 according to which an O/C ratio (as measured with XPS) of 0.5 corresponds to GO and a value between 0.1 and 0.5 would correspond to rGO. That the last material (#9) deviates chemically from GO is also evident in the SEM micrographs in Figure 1, where the adhesion and compactness of the deposited suspension droplet of sample #9 under the same conditions as for the first two GO samples is different. The superior hydrophilicity of GO in comparison to the high hydrophobicity of graphene(‐like) materials is well known. It should be noted that the O/C ratio measured with each method, SEM/EDS and XPS, is also dependent on the information depth of the respective method. It was demonstrated in recent publications of our group on graphene materials which were oxygen‐functionalized by a plasma process [22, 28] that the O/C ratio reflects the preferred functionalization at the outermost particle surfaces. For GO/rGO materials in this study—prepared by a modified Tour's method [29]—a lower O/C ratio was measured with the more surface‐sensitive method, XPS. This lower O/C ratio found by XPS can be explained by the influence of the adventitious carbon [30]. Further, several impurity elements (additionally to O and C as the main elements) are clearly visible in the EDS spectra, see Figure 1e. While material #9 contains only C and O, material #4 contains N and S, and material #7 contains N, Mn and P. To note that the presence of all these impurity elements at the level of roughly 1 at‐% could be explained by the material producer as rests remained from the synthesis.

### 2.2 Commercial GO Material

**FIGURE 2:**
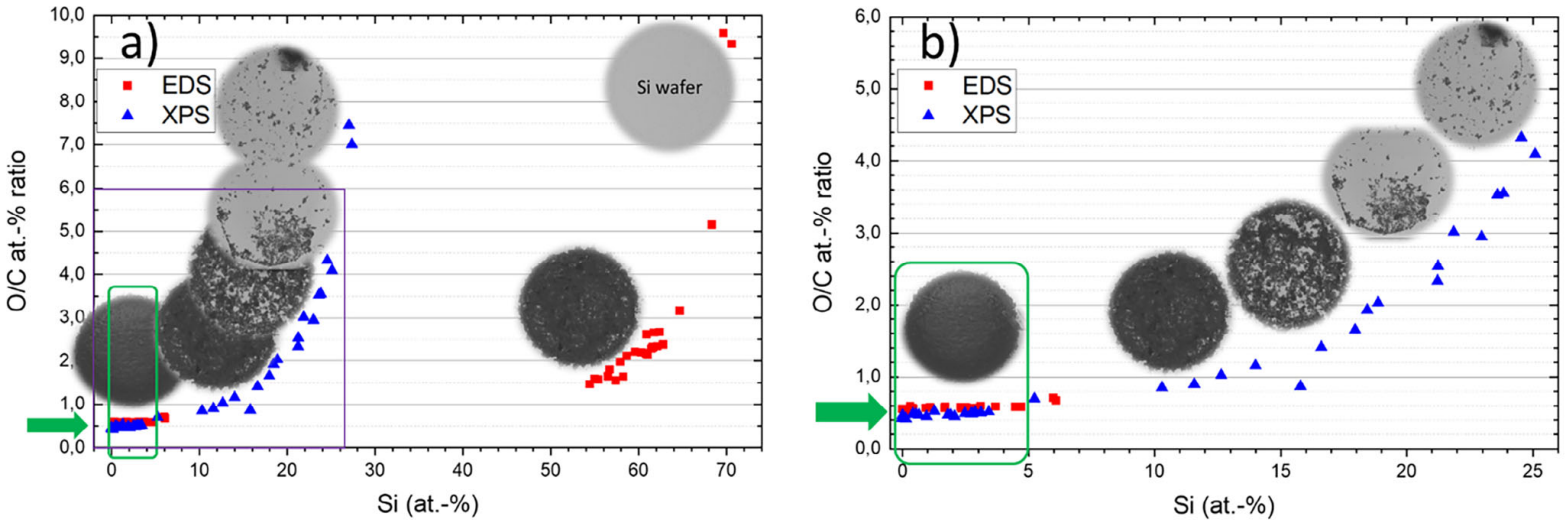
a) O/C at‐% ratio as measured with SEM/EDS (red) and XPS (blue) for a series of GO flakes depositions from liquid suspension onto a silicon substrate having a 70 nm thick SiO_2_ layer on its surface, with the density and thickness of the deposited spots varying from closed spot (no‐substrate visible for analysis, 0 at‐% Si) to very diluted deposited spots, where the Si substrate contribution in spectra is significant, b) zoomed area from a) (see the purple rectangle). The green rectangle denotes the compact and thick depositions, i.e., with a Si substrate contribution below 5 at‐%, which guarantee conditions for accurate both EDS and XPS quantification.

As next, another type of GR2M material has been considered, namely a commercial material containing practically only one‐layer GO flakes in water suspension. The producer offers this material with the following specifications: 52.7 wt‐% C, 43.4 wt‐% O, 1.4 wt‐% H, 0.4 wt‐% N, and 2.1 wt‐% S, i.e., an O/C at% ratio of 0.61. Note that these values were measured as bulk values for this particular batch by elemental analysis after removing the moisture content by TGA. Independent dedicated characterization of this material has recommended this material as particularly suited as a model GR2M sample (with the observation that there is no reference 2D material available), due to its high quality (one‐layer graphene oxide flakes), homogeneity, stability, and also chemical purity. In the following, we report the results of the systematic characterization obtained with metrological SEM/EDS and XPS instruments at BAM under different conditions. One of the main sources of uncertainties contributing to the accuracy of the measurement result is the quality of the sample preparation. A dedicated systematic study is in progress and will be published soon [31]. Figure 2 illustrates the effect of the density/compactness of the drop‐casted deposited GO flake suspension onto a silicon substrate on the O/C at‐% ratio as measured by SEM/EDS at 5 kV and XPS. As expected, only the very compact and thick enough depositions with corresponding spectra containing no significant Si peak provide accurate results, i.e., in the range of a Si contribution of below 5 at‐%, ideally at 0 at‐%. Note the higher sensitivity of XPS to the oxygen present at the surface of the silicon wafer in comparison to EDS, so that the O/C values for XPS are altered (increase) “quicker” with the Si contribution than for EDS. Further, also under compact deposition conditions, the somewhat higher values of O/C atom‐% ratio for EDS in comparison to XPS should be noticed.

**FIGURE 3:**
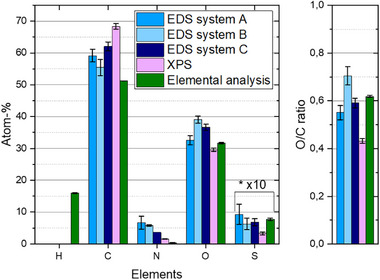
Standardless quantification of the elemental composition of a commercial graphene oxide material by three EDS spectrometers from three different manufacturers (at 5 kV) and XPS after proper sample preparation from liquid suspension on a silicon substrate. The results obtained by elemental analysis have been also added.

The atomic fractions of the elemental composition of this GO material after proper sample preparation, i.e., with a contribution from the silicon substrate below 5 at‐% (see Figure 2), and measurement and quantification with three EDS spectrometers from different manufacturers and one XPS system are showed in Figure 3. It should be noted that the Si substrate (even if with the 70 nm SiO_2_ layer on it) has no contribution as long as the thickness of deposited GO is at least 500 nm, see corresponding Monte Carlo simulations in Figure S12 in Section S6. Further, the values of H, C, N, O and S atomic fractions measured with the conventional elemental analysis method have been also added for comparison, see data in Table S1.

The EDS results for C range from 55.4 to 62.1 at‐%, this means a 12% relative deviation, N varies between 3.6 and 6.7 at‐%, i.e. a 46% relative deviation, O varies between 32.6 and 36.7 at‐%, i.e. a 11% relative deviation, and S yields 0.6 to 0.9 at‐%, i.e. a 33% relative deviation. Comparing now the EDS results to the (reference) XPS ones, the highest differences amounts to −12.9 at‐% for C, +5.1 at‐% for N, +7.2 at‐% for O, and +0.6 at‐% for S. With regard to the O/C at‐% ratio, two EDS spectrometers (A and B) are within 10% relative deviations to each other with an O/C ratio of ≈0.6, and one spectrometer (B) yields a higher value of 0.7. The XPS value of the O/C at‐% ratio is 0.43. Similar to the previous example, this lower O/C ratio can be explained by the presence of the adventitious carbon at the sample surface [30].

High‐resolution XPS spectra of C 1s, O 1s, N 1s, and S 2p have been also taken and are displayed in Figures S2–S6 and Tables S2–S6 together with detailed description and a structural model of GO including six carbon species. Based on the quantitative XPS analysis obtained from the survey spectra, which resulted in an O/C ratio of 0.43, it can be concluded that hydroxyl groups together with peroxide groups dominate, while a significantly smaller fraction of epoxide groups is also present. The quantitative evaluation of the high‐resolution spectra yields an O/C ratio of 0.36, which is lower than the ratio measured in the survey spectra. In general, a lower O/C ratio is observed in the high‐resolution spectra with the spectrometer used, which can be explained by the influence of the transmission function. However, beam damage, in which C─O bonds are broken, cannot be ruled out, see also beam damage discussion later. In this case, hydroxyl or, more likely, peroxide groups would be more affected than epoxide groups.

**FIGURE 4:**
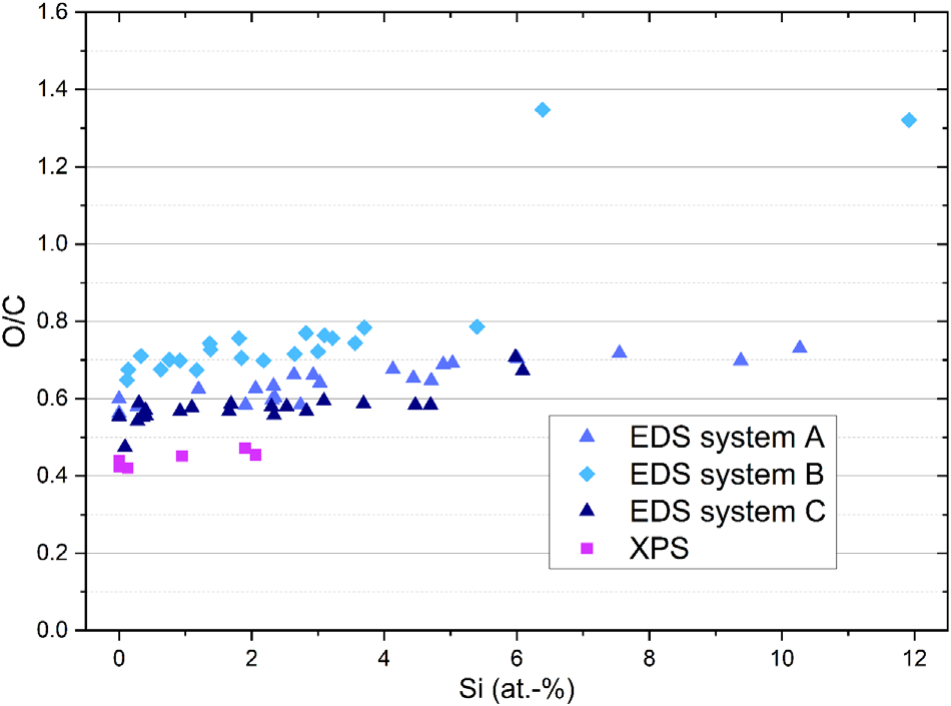
Standardless quantification of the elemental composition of a commercial graphene oxide material by three EDS spectrometers from three different manufacturers (at 5 kV) and XPS for different deposition qualities of sample preparation from liquid suspension on a silicon substrate, i.e., completely compact and thick depositions at 0 at‐% Si as a substrate and increasingly dispersed deposition spots offering more and more Si substrate visible in spectra. Associated measurement uncertainties of 20–30% should be considered for the EDS results and 10–20% for XPS, see further details later and also Table 1.

In Figure 2, the results for the EDS system A have been displayed together with the XPS results. For the sake of completeness, all the results of O/C ratio for the three EDS spectrometers and the XPS system have been collated together in one plot, see Figure 4.

**FIGURE 5:**
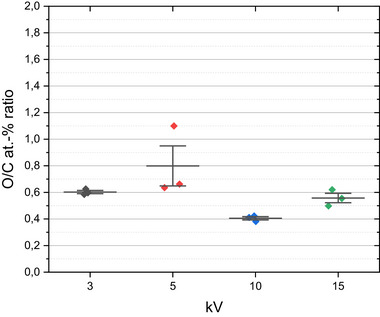
O/C at‐% ratio calculated via standardless EDS analysis at different acceleration voltages, with measurements at same three locations for each kV.

Further, in order to check the robustness of the EDS standardless quantification, four different acceleration voltages, i.e., 3, 5, 10 and 15 kV, have been tested with one EDS spectrometer (EDS system A). The result is represented in Figure 5. Note a variation in the O/C at‐% ratio between 0.4 at 10 kV and 0.65 at 5 kV with an outlier at 1.1. This relatively large deviation makes necessary to always specify in reports the exact acceleration voltage applied. Otherwise, the repeatability of the measurement points at different locations for each individual kV is good, pointing at a good homogeneity of the chemical composition of the commercial GO material as well as a good homogeneity of the sample deposition onto the substrate.

### 2.3 Ionic Liquid as Reference Material for Light Elements

In order to check the validity of both EDS and XPS results after quantification of light elements such as C and O, a model material with a stoichiometric, well‐known elemental composition containing only light elements has been considered. An ionic liquid has been selected as a reference material, due to its commercial availability and convenient price, excellent stability also under vacuum, and homogeneous chemical composition in the whole material volume [24, 25].

**FIGURE 6:**
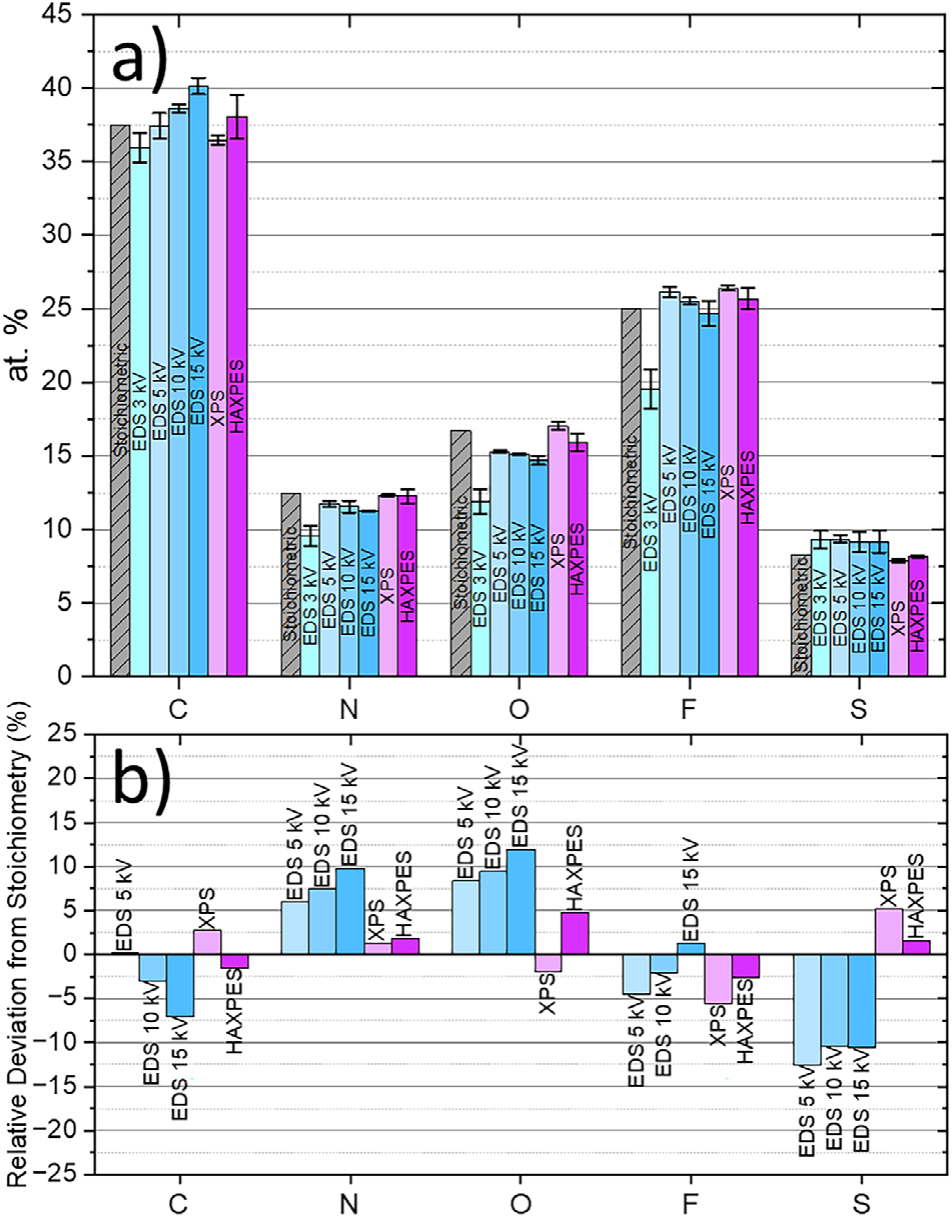
a) Measurement of the elemental composition of an ionic liquid sample selected as a model material by an EDS spectrometer (EDS system C) at 3, 5, 10 and 15 kV and XPS equipped also with HAXPES to validate the quantification of the light elements, in absolute values (at‐%), including the stoichiometric composition as the reference (first gray columns), and b) expressed as relative deviations to the stochiometric composition.

Figure 6a shows the elemental composition (in at‐%) of the ionic liquid after standardless quantification with EDS at 3, 5, 10 and 15 kV (EDS system C) as well as with XPS and HAXPES. The relative deviations to the stoichiometric values are also shown in Figure 6b. Due to the insufficient overvoltage ratio for the excitation of the S K X‐ray lines, which cause a faulty overall quantification, the 3 kV values have been excluded from the representation in Figure 6b.

The lowest relative deviations for all elements are showed by HAXPES, i.e. below 5%, followed by XPS which reports relative deviations slightly above 5% only for F and S (5.6% and 5.2%, respectively). EDS shows relative deviations of about maximum 10% with overestimation of N and O and underestimation of S. EDS quantification of C and F performs well, i.e. below roughly 5%. The other two EDS systems considered in this study (A and B) produce results that deviate by less than 10 at‐% from the stoichiometry for C, N, O and F, with best results for the measurements at 5 kV. These series of measurements clearly validate the capability of all three analytical methods, SEM/EDS, XPS and HAXPES, to quantify light elements in GR2M with an associated measurement uncertainty below 10 rel.‐% for EDS and below 5 rel.‐% for XPS and HAXPES. This excellent experimental proof constitutes the basis for additional upcoming consideration of SEM/EDS (and HAXPES) for the quantification of the elemental composition of GR2M in the existing ISO technical specification TS 23359 where XPS is already the main method defined for this task, and TGA, FTIR and ICP‐MS accompanying it for the amount of impurities and moisture content, for the identification of the functional groups and for the quantification of the trace metal impurities, respectively.

It shall be noted that beam damage may occur for the soft materials in this study, i.e., both GR2M and ionic liquids, if the excitation conditions are improper, see for the SEM/EDS case Section 4 in SI “Experiments on potential beam damage.” The SEM/EDS analysis in the point‐analysis mode shall be avoided for GO, as already after applying a beam current well below 1 nA for 30 s, beam damage becomes clearly visible, notably also at low beam voltages. Therefore, we recommend to scan areas of ≈100 µm^2^. Significant changes in the vacuum during or after the SEM/EDS measurements have not been observed, the vacuum level in the measurement chamber remaining always below 1 × 10^−6^ Pa. As far as XPS is concerned, beam damage can be an issue for GO which can lead to lower O/C ratios within the relative uncertainty range of 10% to 15% range based on our experience. The beam damage depends on the XPS measurement conditions. We recommend the quantification of the survey spectra as well as the minimization of the beam time. Potential beam damage should be taken into account when comparing quantification results from survey and high‐resolution spectra. For the ionic liquid we did not observe any beam damage.

### 2.4 Measurement Uncertainties

#### 2.4.1 SEM/EDS

The deviations from the stoichiometric composition of the ionic liquid sample (for each element C, N, O, F, S and as a sum) were calculated by using various quantification models for the EDS system #B and showed in Tables S8 and S9. The data were also represented in Figure S11. One can conclude that the relative uncertainty due to quantification model applied is max. 5%. Critical point is the extraction of the real background and net peak areas of the X‐ray lines of the light elements C and O and especially N, particularly when the energy resolution of the respective spectrometer is poor.

Further, individual dependencies of the EDS intensities and resulting O/C ratios on various parameters (such as take‐off angle, kV, etc) were systematically carried out based on Monte Carlo simulations and presented in detail in Section S6. “Monte Carlo simulations of the electron trajectories and EDS spectra.” It must be noted that for the absolute intensities of O and particularly C the simulations do not agree well with the measured values. This observation in this very low energy range is in fact expected to be inferior to the much more reliable simulations of the X‐ray line (intensities) at energies above 1 keV. Nevertheless, for analyzing trends when varying different experimental parameters, Monte Carlo simulations are a valuable tool also when working with C K and O K lines and at low excitation voltages. Finally, we can conclude that for the quantitative standardless SEM/EDS analysis of GR2M's uncertainties in the range of max. 10% are observed due to instrumental conditions, between 10% to 20% for sample‐related uncertainties and 10% to 20% for uncertainties in the data treatment procedures, so that an overall uncertainty of 20% to 30% is the result of the investigations in this study, see Table 1.

**TABLE 1:** Analytical figures of merit and main sources of measurement uncertainty at the quantitative analysis of O and C for graphene‐related 2D materials.

	EDS @SEM	XPS
Overall uncertainty (rel.)	20%–30%	10%–20%
Uncertainty: instrumental conditions (rel.)	<10%	<5%
Uncertainty: sample (preparation)‐related (rel.)	10%–20%	10%–15%
Uncertainty: data treatment (rel.)	10%–20%	10%–15%
Limits of detection	≈1 at‐%	0.1–1 at‐%
Information depth	<1 µm (at 5 kV)	≈10 nm
Influence of adventitious carbon	Minor	Significant (<10 at‐% for C)
Influence of surface adsorbates	Negligible	Moderate due to evaporation in UHV
Influence of the substrate	Should be <5 at‐%, ideally substrate element not present in the spectra	Should be <5 at‐%, ideally substrate element not present in the spectra
Charging	Negligible	Charge compensation recommended
Beam damage	May be significant, e.g., in point‐analysis mode	Relative decrease of O/C by <15% is possible in high‐resolution mode

#### 2.4.2 XPS

Based on a recently published insight note, the measurement uncertainty at the quantitative XPS analysis of the same ionic liquid material as that used in the present study can be assigned in the range of 10% to 15% [32]. Considering also the effect of sample preparation, a combined uncertainty of up to 20% can be estimated in general for the analysis of GR2Ms, see Table 1 summarizing further analytical figures of merit.

## 3 Conclusions and Outlook

This study demonstrates the reliability of the standardless quantification of light elements in graphene‐related 2D materials with energy‐dispersive X‐ray spectroscopy (EDS) at a scanning electron microscope (SEM), as a fast and widely available analytical method. For this a set of different laboratory graphene oxide and reduced graphene oxide materials and a high‐quality commercial graphene oxide material were analyzed with standardless SEM/EDS in tandem with X‐ray photoelectron spectroscopy (XPS), including HAXPES capability, as a reference method. GR2M characteristic properties already defined at ISO level such as the oxygen‐to‐carbon at‐% ratio and the impurity concentration [11] have been extracted from the quantification results. Various measurement conditions (including three EDS spectrometers from different manufacturers) and dedicated sample preparation protocols have been tested to ensure accurate results with both methods, EDS and XPS, e.g. sample preparation must guarantee a thick enough and closed deposition of the particulate (flakes) GR2M from the liquid suspension, so that the substrate interference becomes negligible. In line with ISO definitions, O/C ratios of 0.4 to 0.6 corresponding to GO and between 0.1 and 0.5 corresponding to rGO have been found with both methods, with somewhat higher values for EDS. Further, the quantification of the light elements in GR2M with both methods is additionally validated by considering an ionic liquid sample as a reference material containing only C, N, O, F and S. The obtained relative deviations to the stochiometric values are for all 5 elements below 20% for SEM/EDS and below 10% for XPS and HAXPES, i.e. excellent capabilities of all three methods to quantify light elements.

Another positive result of this study is the consideration of the high‐quality GO material as one of the very few available as a commercial material on the market as a suitable candidate for the very first GO reference material with regard to its chemical composition and also morphology.

With the approach combining electron microscopy and EDS it is possible to determine morphology, flake size and elemental composition in one instrument with no transfer in a short time. This can be used not only for raw materials, but it is of great interest for deposited or printed graphene flakes which are used in different applications, like printed or flexible electronics. In one step, the arrangement, size, morphology, and composition of the GR2M can be determined at the same position. The influence of the substrate should be always checked (for the case when the samples are too thin or the deposition is not compact enough).

**FIGURE 7:**
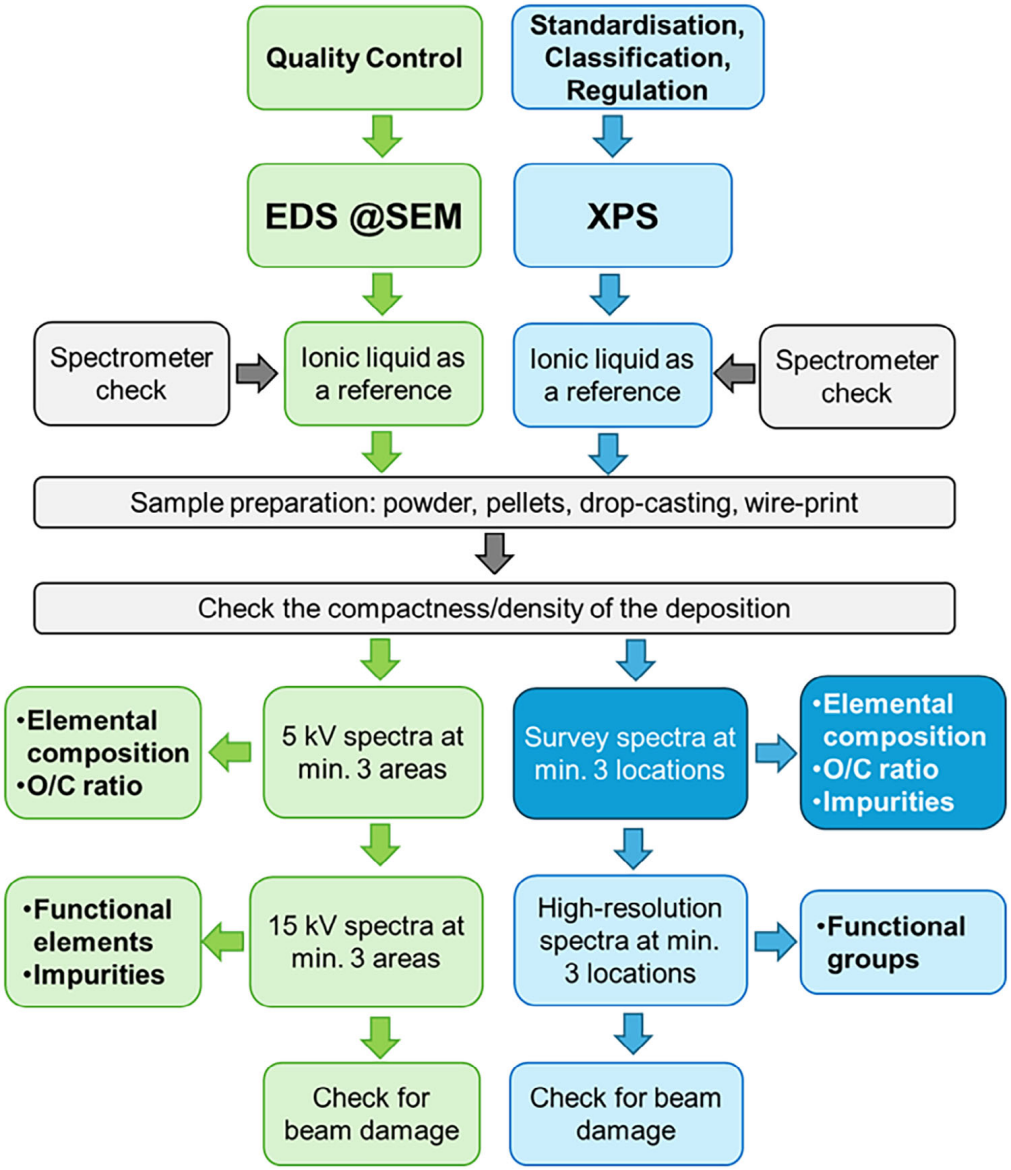
Analysis workflow for EDS @SEM (green) and XPS (blue) measurements of graphene or graphene oxide. The dark‐blue‐filled boxes represent steps standardized in ISO TS 23359:2025 [11].

We are planning to include the knowledge gained in this study with SEM/EDS into standardization, specifically, into ISO/TS 23359 “Nanotechnologies—Chemical characterization of graphene‐related 2D materials from powders and liquid dispersions” [11], adding the dedicated sample preparation [31], with EDS as a reliable quality control method to be applied complementary to XPS as the set method for the chemical analysis of GR2M, see Figure 7. Steps in the overall analysis workflow like spectrometer check (EDS and XPS) by measurement of an ionic liquid as a reference material for light elements, suitable sample preparation (as powder, pellet, or drop‐casting), check of the compactness, thickness and homogeneity of the deposited material on a substrate (preferably silicon wafer), measurement on at least three locations on the sample, e.g., specifically for EDS not in the point‐analysis mode (but scanning an area), use of the proper excitation voltage for EDS (5 and 15 kV, respectively) or application of the high‐resolution mode in XPS, and, finally, check of potential beam damage shall be all carried out carefully. In Table 1 we have summarized the analytical figures of merit at the quantitative analysis of O and C in graphene‐related 2D materials with SEM/EDS and XPS. In the next future, an international interlaboratory comparison exercise to check the broad applicability of the analysis workflow in Figure 7 in the laboratories—as usual for such enterprises—will be conducted [14].

The results presented in this study cover basic and representative GR2M in their raw form, i.e., powder or liquid solution. Studies on the application of the same tandem analytical methods, i.e., SEM/EDS with XPS (and optionally HAXPES), on commercial products containing such raw materials, i.e., inks, just start being published [23]. The additional challenge consists of the small concentration of GR2M material, i.e., in many cases below 1 mass‐% in the product, where other element‐ and volume‐sensitive analytical imaging methods such as ToF‐SIMS (time‐of‐flight secondary ion mass spectrometry) or scanning Auger microscope (SAM) are in advantage [33]. Further, the ‘unfavorable’ chemical composition of the material matrix of low‐loading products (composites, inks, coatings) consisting mainly of oxygen and carbon makes the chemical differentiation between GR2M and matrix material with both EDS and XPS practically impossible. Recent studies have demonstrated that a low fraction of functionalized graphene in inks can be decently analyzed by measuring the key functional element (e.g., F or N) [23, 34]. Last but not least, the actual study contributes significantly to the better understanding of the challenging quantitative structure‐activity relationships of GR2M with more reliable data for the safety and sustainability assessment of this particular category of advanced materials [35, 36].

## 4 Experimental Section/Methods

The GR2M samples have been prepared from the stock solution for analysis by repeated deposition of a drop of liquid suspension onto a silicon wafer to obtain a tight and thicker deposition spot, see Figure 7a. The exact deposition protocol including a systematic study for various nanomaterials and conditions is in the scope of another publication [31]. The ionic liquid was prepared by drop‐casting several tens of microliter into a drilled mm‐cavity (of 5 mm diameter and 3 mm depth) of a conventional aluminum SEM stub, see Figure 7b.

An easy way to assess the quality and homogeneity of the spots deposited onto a substrate is to inspect the spots with a light microscope and apply stylus profilometry to check for the thickness of deposited material. An example is showed in Figure S1 for the commercial GO material, where the compactness of the deposited material can be verified. Further, the thickness of the deposited spot varies radially from about 0.8 µm in the middle down to 0.1 µm at the edge. The (expected) strong local surface morphology is evident. According to Monte Carlo simulations, both with DTSA II and CASINO, we can confirm that a “substrate‐free” SEM/EDS measurement at 5 kV can be reached with a GO deposition thickness of at least 500 nm, see Figure S12.

### 4.1 XPS and HAXPES

Analysis was conducted using a Quantes spectrometer (Ulvac‐PHI), utilizing an Al Kα source emitting at 1.5 keV (XPS) and a Cr Kα source with emitting X‐rays at 5.4 keV (HAXPES). An X‐ray beam spot size of 100 µm, a pass energy of 280 eV (55 eV for high‐resolution measurements), and a step size of 1 eV were chosen for both excitations (XPS and HAXPES). A silicon wafer as the substrate was fixed on a double‐adhesive insulating carbon tape and charge compensation with low‐energy Ar^+^ ions and electrons were used. The spectra were referenced to the main C 1s peak at 284.8 eV. The ionic liquid was filled in a sample holder with a recess which was fixed like the silicon wafer.

**FIGURE 8:**
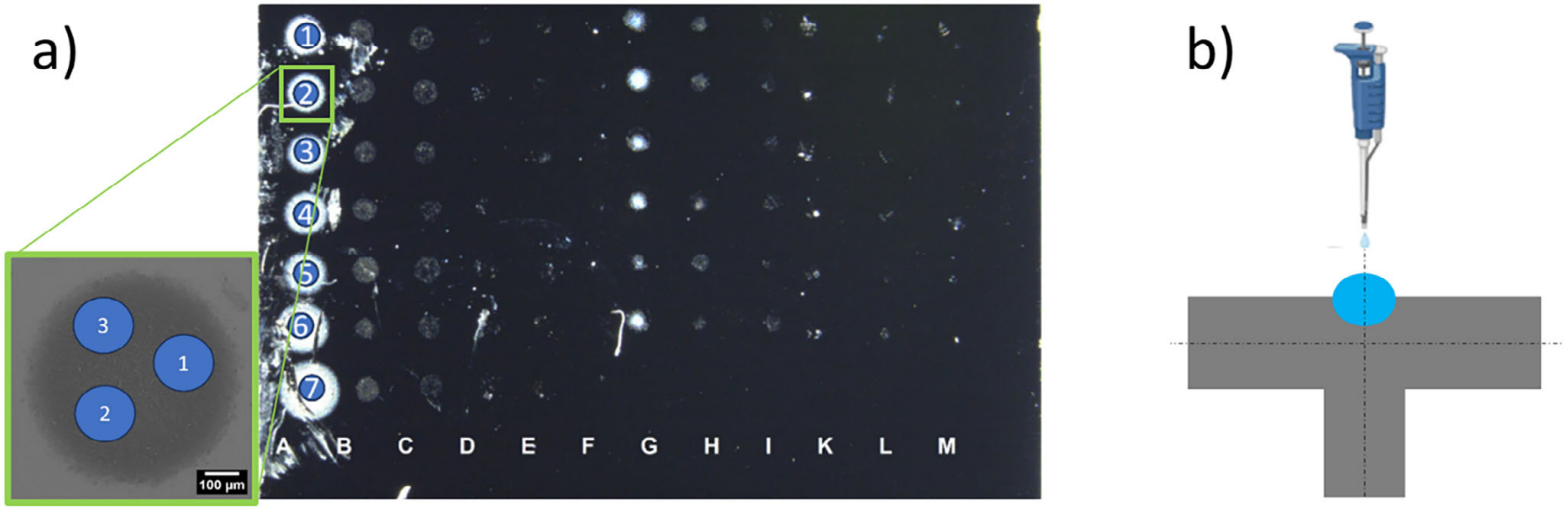
a) Overview of the deposited spots with different GR2M materials onto a 1 cm^2^ silicon wafer under different conditions, with zoomed spot #2 showing three areas where XPS measurements were taken, and b) sketch of the drop‐casting of the ionic liquid into a drilled mm‐cavity of a conventional aluminum SEM stub.

The elemental composition in at‐% is determined from the peak areas after subtracting Shirley backgrounds and using relative sensitivity factors provided by the manufacturer with MultiPak Spectrum:ESCA software (version 9.9.2). For the fitting of the high‐resolution spectra Unifit Version 2024 (Unifit‐Software, Leipzig, Germany) was used with a modified Tougaard‐background [37]. For each sample, each of the deposition spots were measured on three different areas of 100 µm diameter, as sketched in Figure 8a. The mean and standard deviation of the atom% values for each element in each spot were calculated.

### 4.2 SEM/EDS

For the EDS analysis at SEM three spectrometer systems were used: i) An UltraDry SDD detector (ThermoFisher Scientific, active area 100 mm^2^, with analysis software Pathfinder version 1.3, attached to a Zeiss Supra 40 FEG‐SEM), ii) an XFlash 5010 SDD detector (Bruker, active area 10 mm^2^, with an analysis software Esprit version 2.3, attached to the same Zeiss Supra 40 SEM), and iii) an Xplore 30 SDD detector (Oxford, active area 30 mm^2^, analysis software AzTechLight) attached to an SEM of type FlexSEM 1000 II (Hitachi). Each of the deposited spots with different materials were analyzed at 5 kV beam voltage on three circular areas of approximately 100 µm diameter—as depicted in Figure 8a. Deposition spot “1” in the deposition column “A” in Figure 8a was measured at four different beam voltages, i.e. 3, 5, 10 and 15 kV, also at three 100 × 100 µm^2^ areas. Similar to the XPS analysis, reported are the mean values together with their respective standard deviations.

The instrumental and experimental SEM/EDS system‐specific and data analysis parameters have been summarized in Table S7.

## Funding

This project has received funding from the European Union's Horizon Europe Research & Innovation Programme under grant agreement no. 101092796 (ACCORDs — Green deal inspired correlative imaging‐based characterization for safety profiling of 2D materials) and grant agreement no. 101119461 (GrapheneEU). Open Access funding enabled and organized by Projekt DEAL.

## Conflicts of Interest

The authors declare no conflicts of interest.

## Supporting information




**Supporting File**: smll73041‐sup‐0001‐SuppMat.docx.

## Data Availability

The data that support the findings of this study are available from the corresponding author upon reasonable request.
