# Supplementary material for: Standardized Elemental Composition Analysis of Graphene‐Related 2D Materials (GR2M) With SEM/EDS and XPS Works Reliably
Source: Small. 2026 Mar 9;22(25):e11283. doi: 10.1002/smll.202511283 (PMC13137232; doi:10.1002/smll.202511283)
Supplement: Supplementary file 1 — Supporting File: smll73041‐sup‐0001‐SuppMat.docx. [file SMLL-22-e11283-s001.docx]

Supplementary Information

Standardized Elemental Composition Analysis of Graphene-Related 2D Materials (GR2M) with SEM/EDS and XPS Works Reliably

*Paul Mrkwitschka, Mario Sahre, Elena Corrao, Francesco Pellegrino, Beatriz Alonso, Amaia Zurutuza, Jörg Radnik*, and Vasile-Dan Hodoroaba^*^*

P. Mrkwitschka, M. Sahre, J. Radnik, V.-D. Hodoroaba

Division 6.1 Surface and Thin Film Analysis, Federal Institute for Research and Testing (BAM), Berlin, Germany
E-mail: [Joerg.Radnik@bam.de](mailto:Joerg.Radnik@bam.de), [Dan.Hodoroaba@bam.de](mailto:Dan.Hodoroaba@bam.de)

E. Corrao, F. Pellegrino
Department of Chemistry and NIS Centre, University of Torino, Torino, Italy

B. Alonso, A. Zurutuza
Graphenea, San Sebastián, Spain

Table of contents

1. Elemental analysis…………………………………………………………………………...2

2. Profilometry of the deposited sample spots…………………………………………………2

3. High-resolution XPS………………………………………………………………………...3

4. Experiments on potential beam damage…………………………………………………….8

5. SEM/EDS experimental parameters and data-analysis settings……………………………10

6. Monte Carlo simulations of the electron trajectories and EDS spectra……………………13

7. Experimental……………………………………………………………………………….16

8. Acknowledgements………………………………………………………………………...16

9. References………………………………………………………………………………….16

1. **Elemental analysis**

**Table S1.** Elemental analysis (2 measurements, expressed in wt-% and at-%) of the commercial GO material by dynamic flash combustion followed by gas chromatography separation. The resulting O/C ratios have been also added.

| Element | wt-% | | at-% | |
| --- | --- | --- | --- | --- |
|  | measurement #1 | measurement #2 | measurement #1 | measurement #2 |
| H | 1.37 | 1.39 | 15.89 | 16.09 |
| C | 52.68 | 52.7 | 51.26 | 51.19 |
| N | 0.35 | 0.43 | 0.29 | 0.36 |
| O | 43.54 | 43.27 | 31.81 | 31.55 |
| S | 2.07 | 2.21 | 0.75 | 0.80 |
|  |  |  |  |  |
| O/C | 0.82 | 0.83 | 0.62 | 0.62 |

1. **Profilometry of the deposited sample spots**


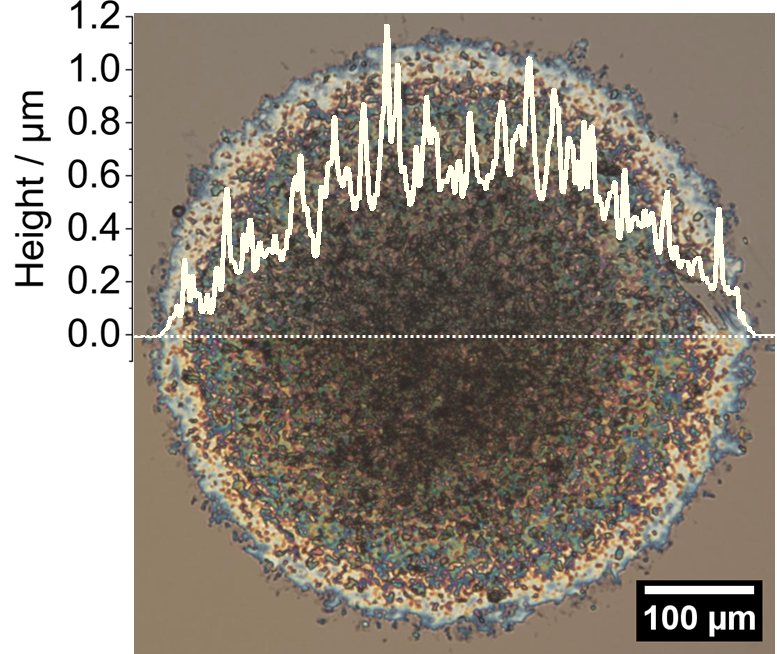


**Figure S1**: Photomicrograph of a deposited spot of graphene oxide (from liquid suspension) together with the profile of the thickness across the middle of the spot.

1. **High-resolution XPS**


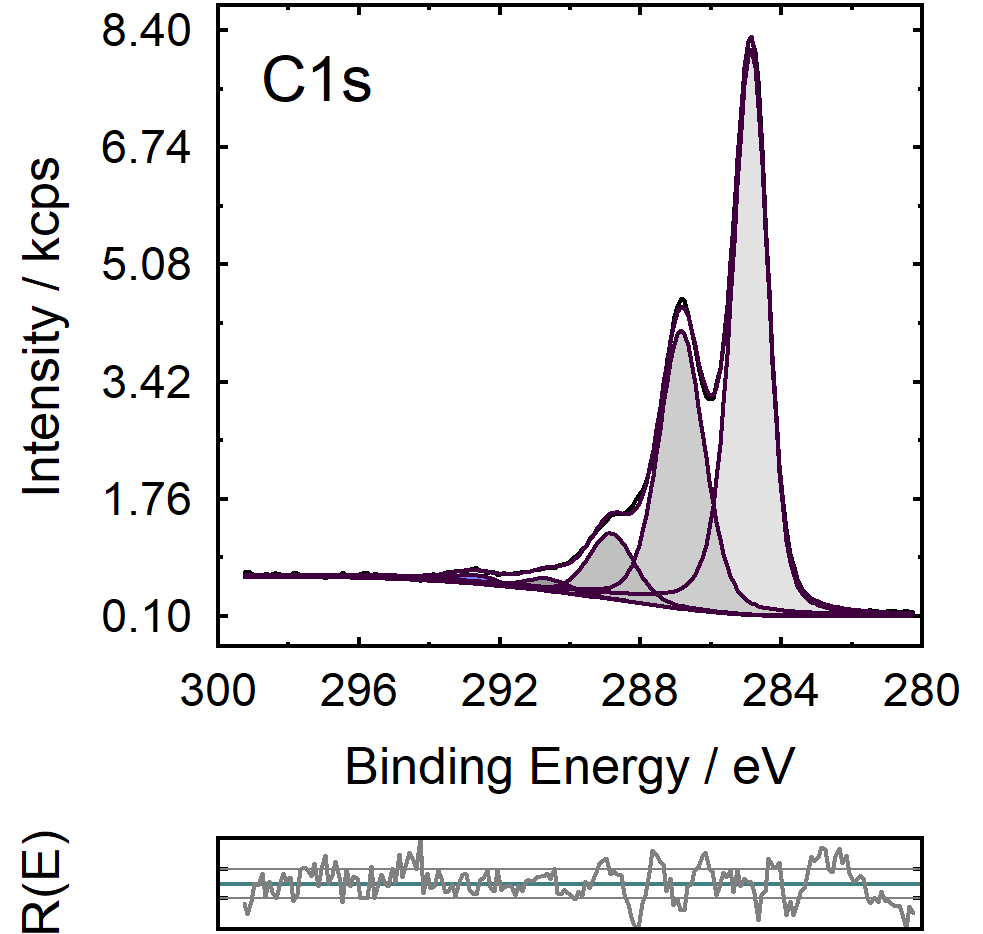


**Figure S2**: High-resolution XPSpectrum of C 1s, including residuals after background subtraction and fitting.

**Table S2**: Fit parameters and results after fitting for the spectrum in Figure S1.


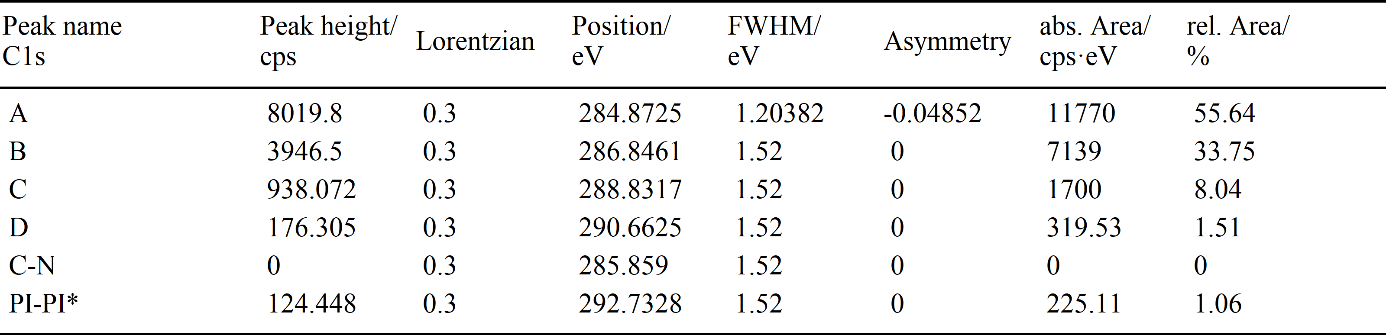


The O/C ratio analysed from the quantitative analysis of the survey spectra was compared with the results which can be obtained from the high-resolution spectra. This evaluation was conducted in accordance with a recently published study on the calculation of the oxygen content in adventitious carbon, derived from high-resolution C 1s spectra [1]. Assuming the basic structural model of graphene oxide (Figure S2), five distinct peaks can be expected, which can be correlated with the various carbon species found in graphene oxide, see Table S2. For each of these species, the O/C ratio is known.


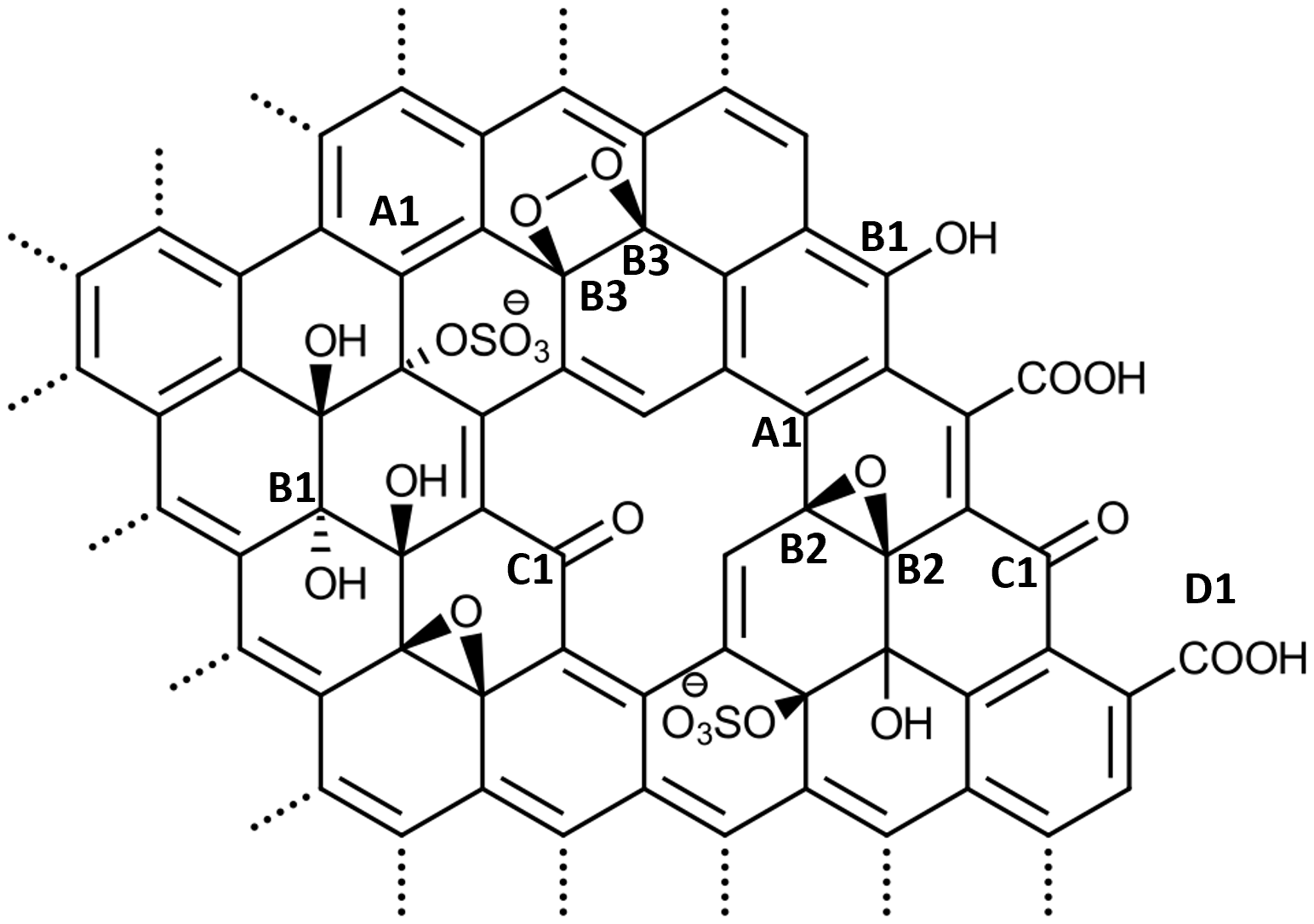


**Figure S3**: Structural model of graphene oxide. The different species are labelled as follows: A1 (aromatic C-C or C-H), B1 (hydroxyl groups), B2 (epoxide groups), B3 (peroxide groups), C1 (carbonyl groups) and D1 (carboxyl groups).

**Table S3**: The peaks found in the high-resolution spectra, the carbon species (see explanations in the caption of Figure S3) and the corresponding O/C ratios.

| **Peak** | **Species** | **O/C ratio** |
| --- | --- | --- |
| A | A1 | 0 |
| B | B1 | 1 |
|  | B2 | 0.5 |
|  | B3 | 1 |
| C | C1 | 1 |
| D | D1 | 0.5 |
| C-N | C-N species | 0 |
| π-π* | A1 | 0 |

The ratio between B2 and B1/B3 remains unclear. As a first approximation, one may assume that the number of epoxide groups (B2) equals that of hydroxyl (B1) and peroxide (B3) groups. Together with the area obtained from the fitting for the peaks C and D, this results in an O/C ratio of 0.33. If, however, it is assumed that no epoxide groups are present, the resulting O/C ratio would be 0.49. Minor amounts of oxygen, below 1 at-%, are likely bound to sulfur and to traces of silicon; these contributions are not considered further. Due to the high-resolution N 1s spectra, the presence of oxidized N species can be excluded. Based on the quantitative XPS analysis obtained from the survey spectra, which resulted in an O/C ratio of 0.43, it can be concluded that hydroxyl groups together with peroxide groups dominate, while a significantly smaller fraction of epoxide groups is also present.

The quantitative evaluation of the high-resolution spectra yields an O/C ratio of 0.36, which is lower than the ratio measured in the survey spectra. In general, a lower O/C ratio is observed in the high-resolution spectra with the spectrometer used, which can be explained by the influence of the transmission function. However, beam damage, in which C-O bonds are broken, cannot be ruled out. In this case, hydroxyl or, more likely, peroxide groups would be more affected than epoxide groups.


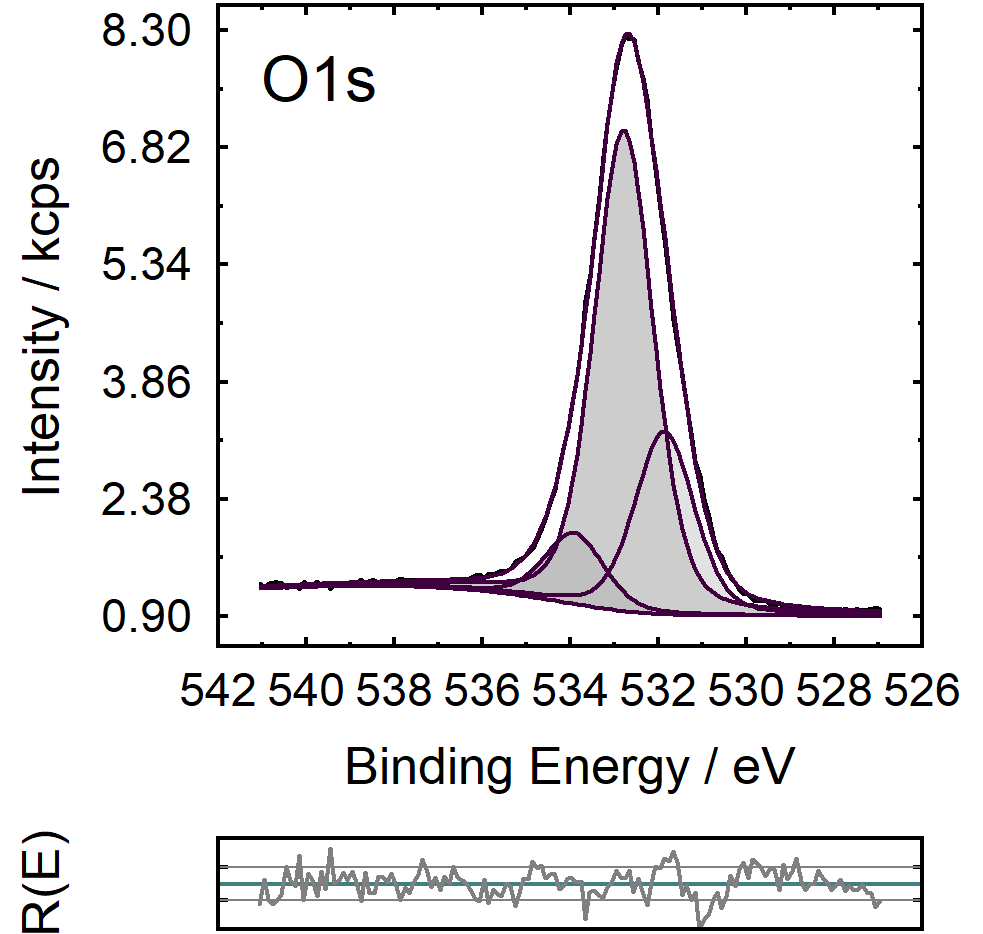


**Figure S4**: High-resolution XPSpectrum of O 1s, including residuals after background subtraction and fitting.

**Table S4**: Fit parameters and results after fitting for the spectrum in Figure S4.


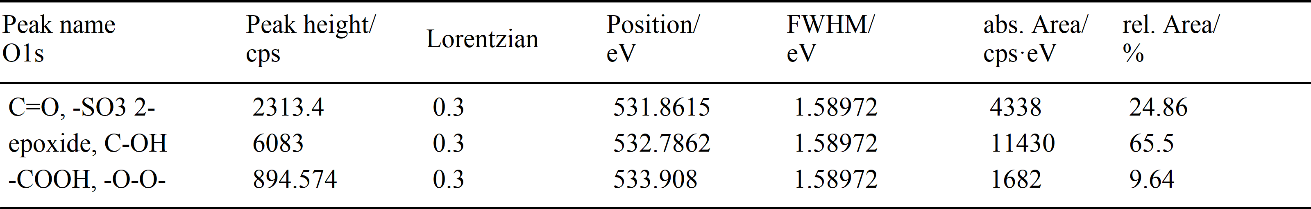


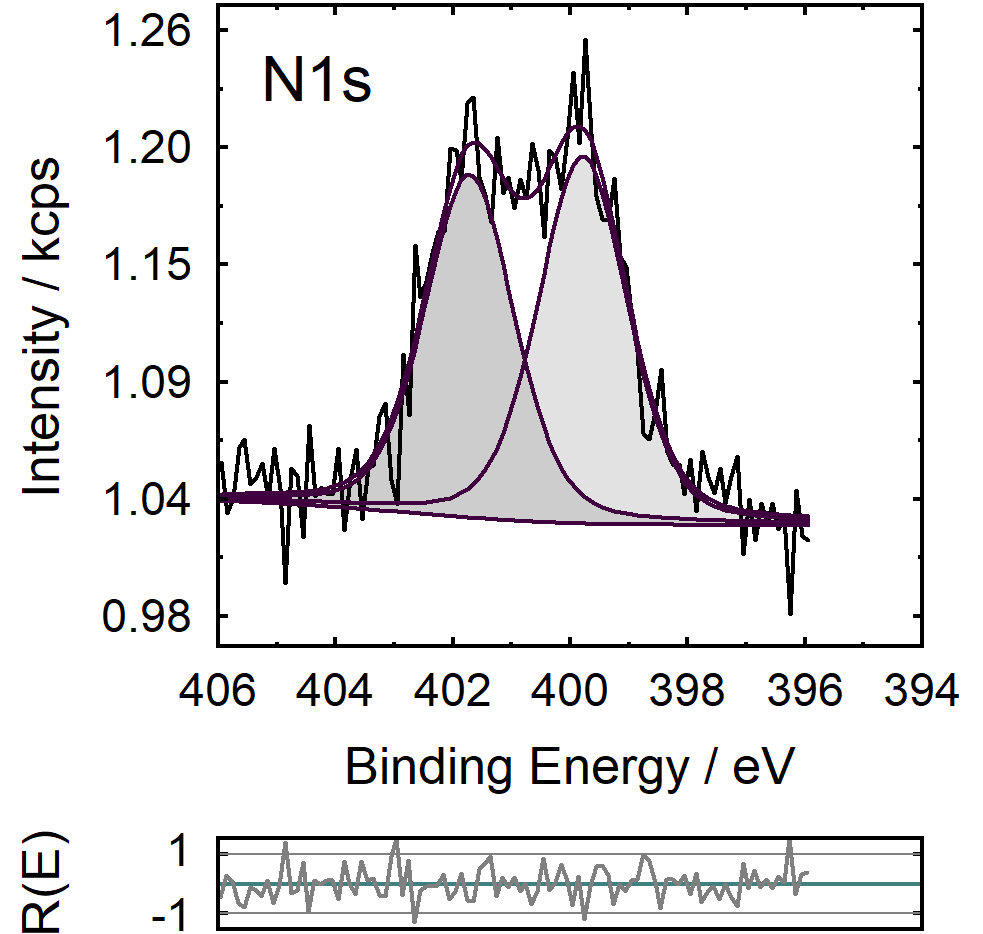


**Figure S5**: High-resolution XPSpectrum of N 1s, including residuals after background subtraction and fitting.

**Table S5**: Fit parameters and results after fitting for the spectrum in Figure S5.


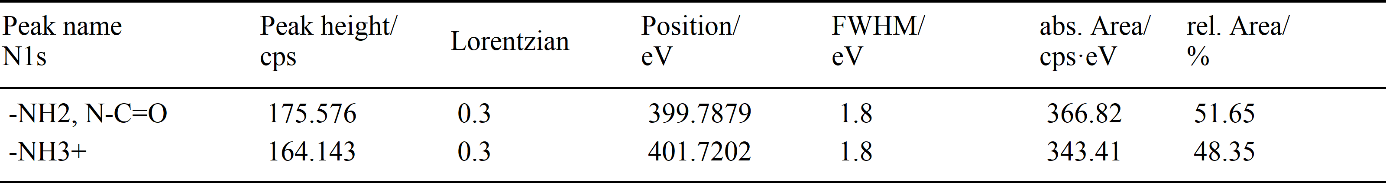


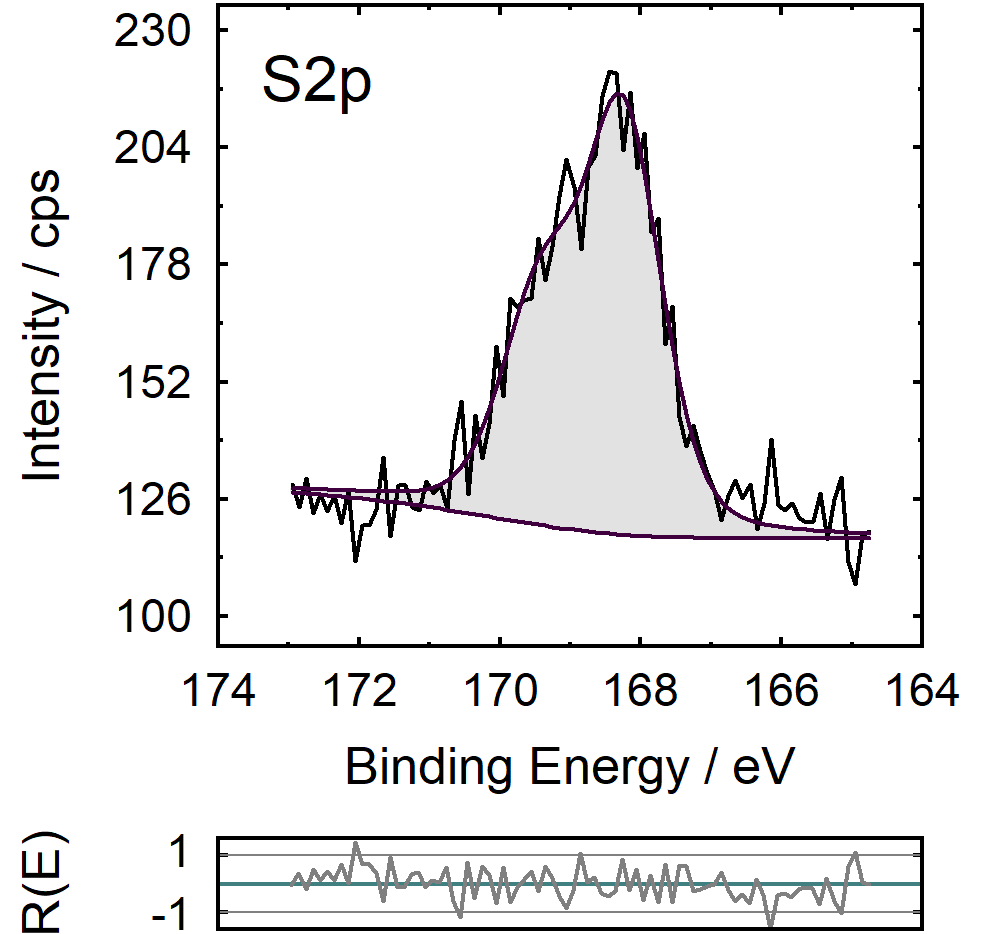


**Figure S6**: High-resolution XPSpectrum of S 2p, including residuals after background subtraction and fitting.

**Table S6**: Fit parameters and results after fitting for the spectrum in Figure S6.


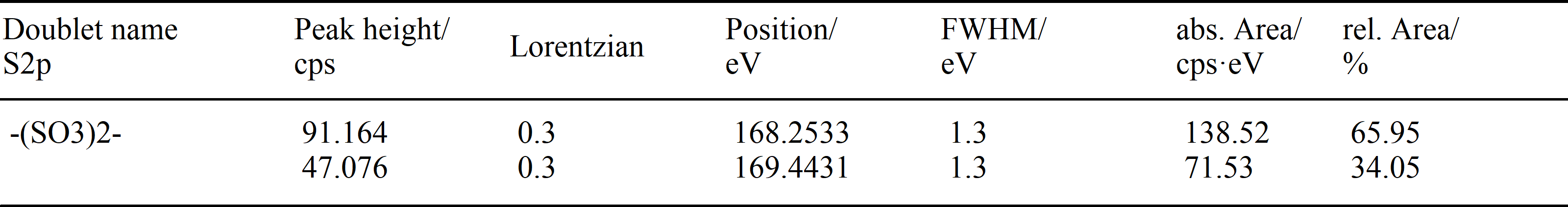


1. **Experiments on potential beam damage**


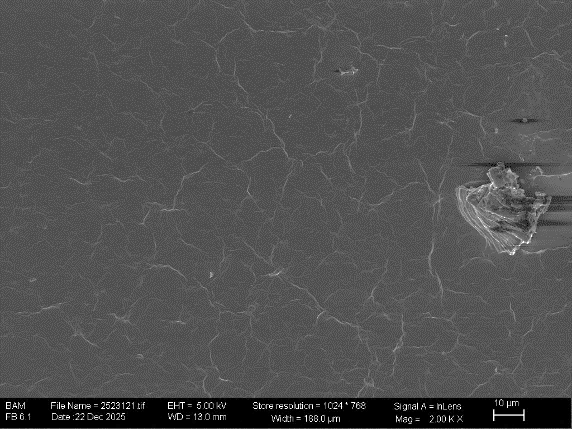

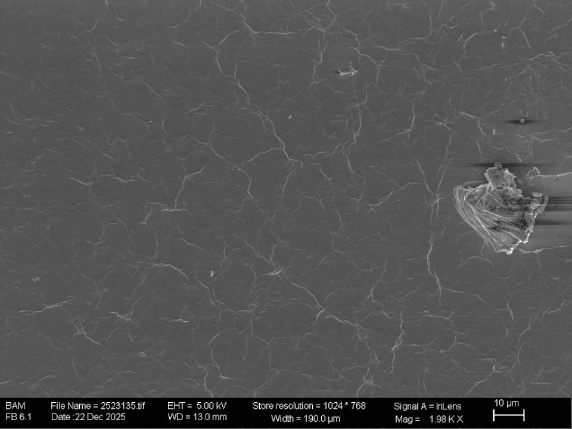


Dosis: 0.16 nA x 500 s @5 kV

Mode: area

Vacuum: unchanged

0 s

10 µm

**Figure S7:** SEM micrographs of graphene oxide before and after an EDS measurement in the scan mode, with a beam current of 0.16 nA, at 5 kV beam voltage.


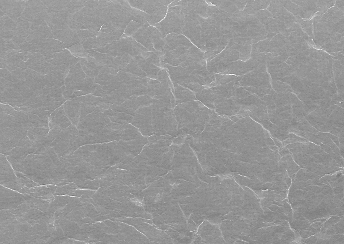

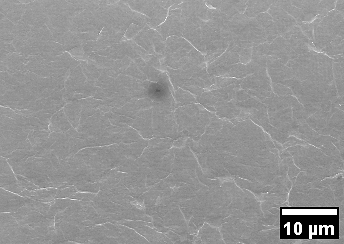


Dosis: 0.16 nA x 100 s @5 kV

Mode: point

Vacuum: unchanged

0 s

**Figure S8:** SEM micrographs of graphene oxide before and after an EDS measurement in point-analysis mode, with a beam current of 0.16 nA, at 5 kV beam voltage.


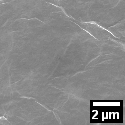

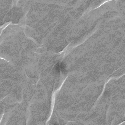

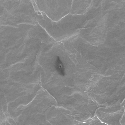


Dosis: 0.16 nA x 30 s, @3 kV

Mode: point

Vacuum: unchanged

Dosis: 0.16 nA x 30 s, @5 kV

Mode: point

Vacuum: unchanged

Dosis: 0.16 nA x 100 s, @15 kV

Mode: point

Vacuum: unchanged

**Figure S9:** SEM micrographs of graphene oxide after EDS measurements in point-analysis mode, with a beam current of 0.16 nA and at different beam voltages: 3, 5 and 15 kV.


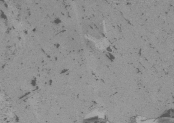

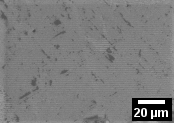


Dosis: 0.16 nA x 100 s, @5 kV

Mode: area

Vacuum: unchanged

0 s

**Figure S10:** SEM micrographs of the ionic liquid sample before and after an EDS area measurement, with a beam current of 0.16 nA, and at 5 kV beam voltage.

**Table S6:** SEM/EDS study on experimental conditions with observations targeted to evaluate potential beam damage for graphene oxide and ionic liquid samples; red= critical conditions, orange=partially critical conditions, yellow= slight changes observed, green= stable conditions (no changes).

| **Graphene Oxide** | | | |
| --- | --- | --- | --- |
| Beam current: 0.16 nA | | | |
| Exposure time, Point/Scan mode | Acceleration voltage | | |
|  | 3 kV | 5 kV | 15 kV |
| 100 s, 100 µm x 100 µm | Darkening visible | Slight discoloration visible | No change of the surface visible |
| 1 s, point | Slight darkening visible | Slight darkening visible | No change visible |
| 30 s, point | Small hole of first few layers and visible darkening | Visible black spot at probed surface |  |
| 100 s, point | n/A | n/A | Faint darkening of probed surface |
| Beam current: >1 nA | | | |
|  | 3 kV | 5 kV | 15 kV |
| 100 s, 100 µm x 100 µm | Darkening visible | | |
| 1 s, point | Obsolate (due to tendency) | | |
| 30 s, point | Obsolate (due to tendency) | | Stable vacuum/ visible hole/crater in the sample |
| 100 s, point |  |  |  |
|  |  | |  |
| **Ionic Liquid** | | | |
| Probe current: 0.16 nA | | | |
| Exposure time, Point/Scan mode | Acceleration voltage | | |
|  | 3 kV | 5 kV | 15 kV |
| 1-30 s, 100 µm x 100 µm | n/A | | |
| 30-100 s, 100 µm x 100 µm | Obsolate | Darkening visible | Slight darkening and movement of the fluid „crust“ |
| 130-200 s, 100 µm x 100 µm | n/A | Crust is dissolved | Strong darkening and visible change of the probed surface |
|  | | | |
| **Conclusion** | | | |
| SEM chamber vacuum | Stable, very minor changes observed in the range of 2x10^-5^ Pa, with no conclusive evidence of either surface change. | | |
| Influence on elemental composition | No significant changes/trends observed. | | |

1. **SEM/EDS experimental parameters and data-analysis settings**

**Table S7:** Instrumental and experimental SEM/EDS system-specific and data analysis parameters.

|  | **EDS #A** | **EDS #B** | **EDS #C** |
| --- | --- | --- | --- |
| WD /mm | 10 | 13 | 13 |
| Probe current /nA | <1 nA | 0.16 | 1.00 |
| Elevation angle | 29 | 35 | 35 |
| Detector (nominal) area /mm^2^ | 30 | 100 | 10 |
| Slide Position /mm | ~27 | 50 | 50 |
| Detector solid angle /msr | ~30 | ~40 | ~4 |
| Window | AP3.3 | AP3.3 | AP3.3 |
| Front contact | Ni 10 nm | Al 30 nm | Ni 10 nm |
| Energy resolution @Mn Kα /eV | 131 | 131 | 125 |
| Live time /s | 40 | 100 | 400 |
| Dead time | <5% | <5% | <5% |
| Quantification model | Phi(rho-Z) (XPP) | Phi(rho-Z) (PROZA) | Phi(rho-Z) (PROZA) |
| Background model | Filtered Least-Squares Fitting (FLS) | FLS | Manually fitted |
| Peak fitting | FLS | FLS | Series fit |

Table S8: Deviations from the stoichiometric composition of the ionic liquid sample (for each element C, N, O, F, S and as a sum) calculated by using various quantification models for the EDS system #3. See representation of the data in Figure S11.

|  | (Absolute) Deviation from stoichiometry  (atom-%) | | | | | Relative deviation from stoichiometry (%) |
| --- | --- | --- | --- | --- | --- | --- |
|  | Mean | SD | Sum | Min | Max | Sum |
| Series-Fit  ZAF+PhiRhoZ  SEM | 1.5 | 0.8 | 7.5 | 0.27 (N) | 2.33 (C) | 40.0 |
| Fit  ZAF+PhiRhoZ  SEM | 1.5 | 0.9 | 7.7 | 0.25 (N) | 2.47 (C) | 40.6 |
| Bayes Entfaltung  ZAF+PhiRhoZ  SEM | 1.7 | 1.1 | 8.7 | 0.21 (N) | 3.06 (C) | 45.7 |
| Series-Fit  PhiRhoZ  SEM | 0.5 | 0.3 | 2.4 | 0.21 (S) | 0.77 (N) | 14.2 |
|  | Recommended by vendor | | | | | |
| Fit  PhiRhoZ  SEM | 0.5 | 0.2 | 2.7 | 0.25 (O) | 0.78 (N) | 15.7 |
| Bayes Entfaltung  PhiRhoZ  SEM | 0.8 | 0.5 | 4.0 | 0.11 (S) | 1.37 (C) | 21.6 |
| Series-Fit  ZAF+PhiRhoZ  Mathe | 1.6 | 0.6 | 7.9 | 0.92 (S) | 2.56 (F) | 45.3 |
| Fit  ZAF+PhiRhoZ  Mathe | 1.7 | 0.6 | 8.3 | 1.01 (S) | 2.69 (F) | 47.4 |
| Bayes Deconvolution  ZAF+PhiRhoZ  Mathe | 0.3 | 0.2 | 1.3 | 0.07 (O) | 0.51 (S) | 11.4 |
| Series-Fit  PhiRhoZ  Mathe | 0.3 | 0.2 | 1.5 | 0.04 (O) | 0.50 (N) | 11.5 |
| Fit  PhiRhoZ  Mathe | 0.3 | 0.2 | 1.3 | 0.07 (O) | 0.51 (S) | 11.4 |
| Bayes Deconvolution  PhiRhoZ  Mathe | 0.3 | 0.3 | 1.4 | 0 (F) | 0.71 (N) | 12.7 |





**Figure S11:** Visualized data from Table S8.

Table S9: Minimum and maximum (relative) deviations from the stoichiometric composition of the ionic liquid calculated with different EDS quantification models for the EDS system #3.

| Element | Minimum relative deviation (%) |  | Maximum relative deviation (%) |  |
| --- | --- | --- | --- | --- |
| C | 0.03 | Bayes Deconvolution  PhiRhoZ  Mathe | 8.2 | Bayes Deconvolution  ZAF+PhiRhoZ  SEM |
| N | 1.7 | Bayes Deconvolution  ZAF+PhiRhoZ  SEM | 11.3 | Series-Fit  ZAF+PhiRhoZ  Mathe |
| O | 0.2 | Series-Fit  PhiRhoZ  Mathe | 11.9 | Bayes Deconvolution  ZAF+PhiRhoZ  SEM |
| F | 0 | Bayes Deconvolution  PhiRhoZ  Mathe | 10.8 | Fit  ZAF+PhiRhoZ  Mathe |
| S | 1.3 | Bayes Deconvolution  PhiRhoZ  SEM | 15.4 | Bayes Deconvolution  ZAF+PhiRhoZ  SEM |

1. **Monte Carlo simulations of the electron trajectories and EDS spectra**

First, we have simulated the electron trajectories in a 1 µm thick graphene oxide sample deposited on a 72 nm SiO_2_-coated silicon wafer at different beam voltages, see Figure S12. It is evident that a „substrate-free“ SEM/EDS measurement at 5 kV can be reached with a GO deposition thickness of at least 500 nm.


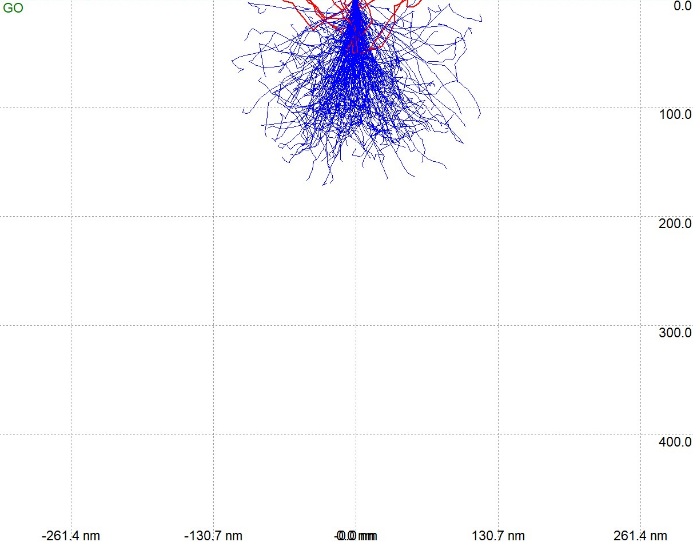

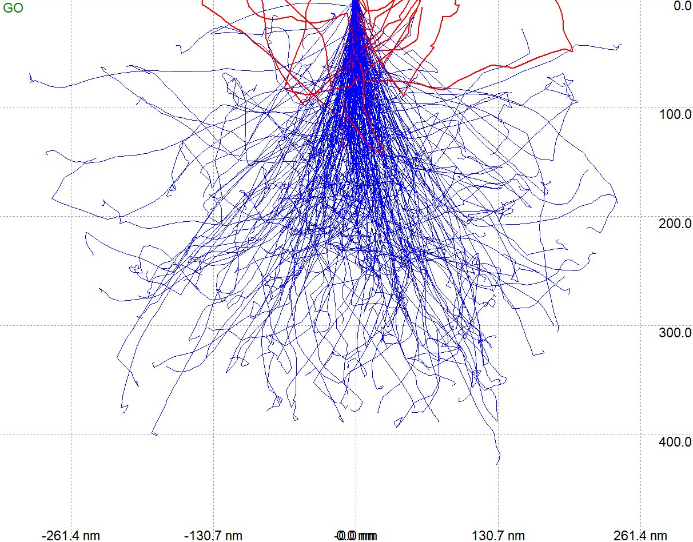

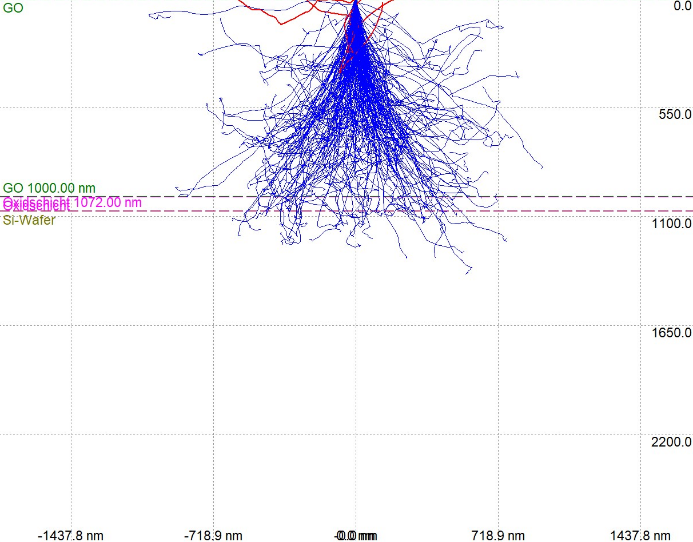

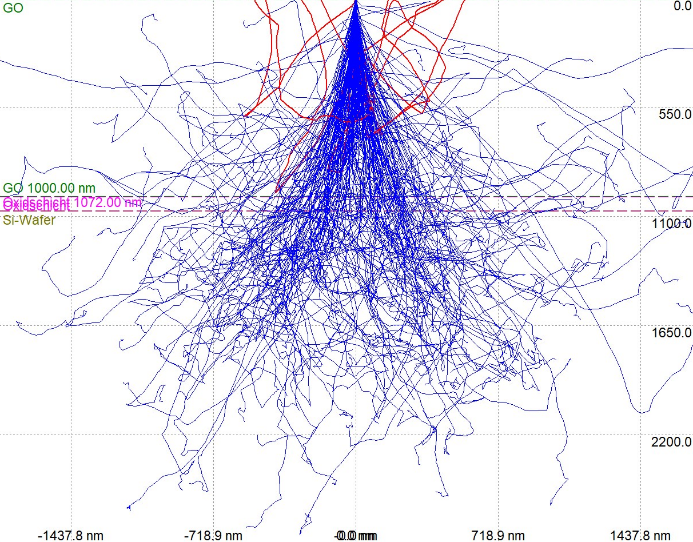


**Figure S12:** Monte Carlo simulation of electron trajectories in a 1 µm thick graphene oxide sample deposited on a 72 nm SiO_2_-coated silicon wafer, at 3 kV, 5 kV, 10 kV and 15 kV beam voltage. Note the two different length scales at 3 and 5 kV (top figures) and 10 and 15 kV (bottom figures).

Generated and emitted C K and O K X-ray intensities from graphene oxide, at different kV were simulated with Casino v2.42 and DTSA II, and the O/C ratio of the emitted photons have been extracted, see Tables S10 and S11. Note the significantly higher O/C ratios at low voltages when the DTSA II software is applied.

**Table S10:** Generated and emitted C K and O K X-ray intensities from graphene oxide, at different kV simulated with Casino v2.42, together with the extracted O/C ratio of the emitted photons.

|  | **C** | **O** | **O/C** |
| --- | --- | --- | --- |
| **Beam voltage /keV** | **Photons: generated/emitted** | | **emitted/emitted** |
| 3 | 202/185 | 232/201 | 1.1 |
| 5 | 415/332 | 542/380 | 1.1 |
| 10 | 929/473 | 1430/483 | 1.0 |
| 15 | 734/351 | 1284/371 | 1.1 |

**Table S11:** Generated and emitted C K and O K X-ray intensities from graphene oxide, at different kV simulated with SX DTSA II, together with the extracted O/C ratio of the emitted photons.

|  | **C** | **O** | **O/C** |
| --- | --- | --- | --- |
| **Beam voltage /keV** | **Photons: generated/emitted** | | **emitted/emitted** |
| 3 | 510/577 | 746/945 | 1.5 |
| 5 | 855/1162 | 1236/2205 | 1.5 |
| 10 | 1053/2689 | 1179/5775 | 1.1 |
| 15 | 837/ 4250 | 851/9577 | 1.0 |

The comparison of a measured EDS spectrum with a simulated one (DTSA II) is shown in Figure S13 for the example of the reference ionic liquid sample. Note the large discrepance of the C K intensity, i.e. the much lower intensity in the simulated spectrum.

**Figure S13:** Comparison of the measured EDS spectrum of the reference ionic liquid sample (5 kV, EDS #B) with the Monte Carlo (DTSA II) simulated spectrum for the same conditions (left) and normalized (right).

We have simulated various conditions. No significant alteration of the O/C intensity ratio for the ionic liquid sample is noticed when the take-off angle changes (between 25° and 45°), see Figure S14. A significant change in the O/C ratio is observed when the beam voltage is varied, see Figure S15. We recommend the use of 5 kV.

**Figure S14:** 5 kV EDS intensities of the C K, N K, O K, F K and S Kα lines corresponding to the ionic liquid sample calculated by Monte Carlo simulations (DTSA II Oberon) and compared with the measurement at different take-off angles. Note that the simulated values are net count intensities, the measured values are net peak areas.

**Figure S15:** EDS intensities of the C K, N K, O K, F K and S Kα lines corresponding to the ionic liquid sample calculated by Monte Carlo simulations (DTSA II Oberon) at different beam voltages.

1. **Experimental**

**Profilometry** measurements were conducted on a DektakXT (Bruker GmbH) with a stylus radius of 2 µm, applying 1 mg force with a resolution of 0.266 µm per point.

Light microscopy imaging was performed on a Polyvar-Met microscope (Reichert-Jung, Wien).

**Elemental analysis** was carried out on a CHNS 2400 Series II (Perkin Elmer) device utilizing the Dumas principles of Dynamic Flash Combustion followed by gas chromatography separation of the resultant gaseous species (N_2_, CO_2_, H_2_O_2_, SO_2_) and TCD detection.

**Acknowledgments**

We thank Ms. Deniz Hülagü (BAM) for providing ellipsometry measurements and Mr. Matthias Weise (BAM) for performing profilometry measurements.

References

[1] J. D. Henderson, B. P. Payne, N. S. McIntyre, M. C. Biesinger, Enhancing Oxygen Spectra Interpretation by Calculating Oxygen Linked to Adventitious Carbon, *Surf. Interface Anal.* 2025, *57*, 214-220, https://doi.org/10.1002/sia.737.
